# John Charles Steele

**Published:** 1892-11-26

**Authors:** 


### IN MEMORIAM.

### JOHN CHARLES STEELE, M.D.

For forty years Medical Superintendent of Guy's Hospital.

And he is gone! The man of honest heart,
Whose voice for truth and right was ever heard,
The man who ever chose the better part,
Nor spoke an unkind word.

How we shall miss the dear benignant face
We knew so well within the grey old square.
Guy's will appear to all another place,
If he may not be there.

Calmly he sees the evening shadows fall,
The labourer rests at setting of the sun.
He hears—we cannot hear—the Master's call
"Servant of God, well done."

Around thy grave the weeping mourners stand
In grief bowed down. Ah! What shall break the spell?
Accept this heartfelt tribute from the hand
Of one who loved thee well.

